# Genetic and Genomic Characterization of a New Beef Cattle Composite Breed (Purunã) Developed for Production in Pasture-Based Systems

**DOI:** 10.3389/fgene.2022.858970

**Published:** 2022-07-18

**Authors:** Henrique Alberto Mulim, Luiz F. Brito, Luís Fernando Batista Pinto, José Luis Moletta, Lilian Regina Da Silva, Victor Breno Pedrosa

**Affiliations:** ^1^ Department of Animal Science, Federal University of Bahia, Salvador, Brazil; ^2^ Department of Animal Sciences, Purdue University, West Lafayette, IN, United States; ^3^ Agronomic Institute of Paraná—IAPAR, Ponta Grossa, Brazil; ^4^ NEOGEN Corporation, Pindamonhangaba, Brazil; ^5^ Department of Animal Science, State University of Ponta Grossa, Ponta Grossa, Brazil

**Keywords:** beef cattle, genomic diversity, inbreeding coefficient, persistency of the gametic phase, runs of heterozygosity, runs of homozygosity

## Abstract

Purunã is a composite beef cattle breed, developed in Southern Brazil by crossing the Angus, Charolais, Canchim, and Caracu breeds. The goal of this study was to perform the first genetic characterization of the Purunã breed, based on both pedigree and genomic information. For this, 100 randomly selected animals were genotyped, and 11,205 animals born from 1997 to 2019 had pedigree information. The genetic analyses performed were principal component analysis, admixture, phylogenic tree, pedigree and genomic inbreeding, linkage disequilibrium (LD), effective population size (Ne), consistency of the gametic phase, runs of homozygosity (ROH), heterozygosity-enriched regions (HERs), and functional analyses of the ROH and HER regions identified. Our findings indicate that Purunã is more genetically related to the Charolais, Canchim, and Angus breeds than Caracu or Nellore. The levels of inbreeding were shown to be small based on all the metrics evaluated and ranged from −0.009 to 0.029. A low (−0.12–0.31) correlation of the pedigree-based inbreeding compared to all the genomic inbreeding coefficients evaluated was observed. The LD average was 0.031 (±0.0517), and the consistency of the gametic phase was shown to be low for all the breed pairs, ranging from 0.42 to 0.27 to the distance of 20 Mb. The Ne values based on pedigree and genomic information were 158 and 115, respectively. A total of 1,839 ROHs were found, and the majority of them are of small length (<4 Mb). An important homozygous region was identified on BTA5 with pathways related to behavioral traits (sensory perception, detection of stimulus, and others), as well as candidate genes related to heat tolerance (*MY O 1A*), feed conversion rate (*RDH5*), and reproduction (*AMDHD1*). A total of 1,799 HERs were identified in the Purunã breed with 92.3% of them classified within the 0.5–1 Mb length group, and 19 HER islands were identified in the autosomal genome. These HER islands harbor genes involved in growth pathways, carcass weight (*SDCBP*), meat and carcass quality (*MT2A*), and marbling deposition (*CISH*). Despite the genetic relationship between Purunã and the founder breeds, a multi-breed genomic evaluation is likely not feasible due to their population structure and low consistency of the gametic phase among them.

## 1 Introduction

The characterization of the population structure and genetic diversity is essential for the understanding of the genetic background of environmental adaptation and conservation of cattle genetic resources (Xia et al., 2021). Such characterization and diversity assessment need to be considered when designing or updating breeding programs and conservation strategies that can be applied in purebred and crossbred populations.

The Purunã breed is a composite population developed in Southern Brazil by crossing Angus, Charolais, Canchim, and Caracu, in identical proportions. This was performed to improve key traits of interest and exploit the complementarity among the breeds (Otto et al., 2021), especially for production in pasture-based systems. The background research to generate the Purunã breed started at the Agronomic Institute of Paraná (IAPAR; Ponta Grossa, Paraná, Brazil) at the beginning of the 1980s, when IAPAR researchers estimated the heterosis in the crossbred progenies of Charolais x Caracu and Angus x Canchim (Perotto et al., 2000a; Perotto et al., 2000b). Almost 15 years later, the first results were obtained from this experiment, where the heterosis retained from those crosses resulted in higher hot carcass weight, hot carcass yield, rib-eye area, better carcass conformation from Charolais x Caracu (Perotto et al., 2000b), and higher average weight daily gain in different ages with the crosses of Angus x Canchim (Perotto et al., 2000a).

**FIGURE 1:**
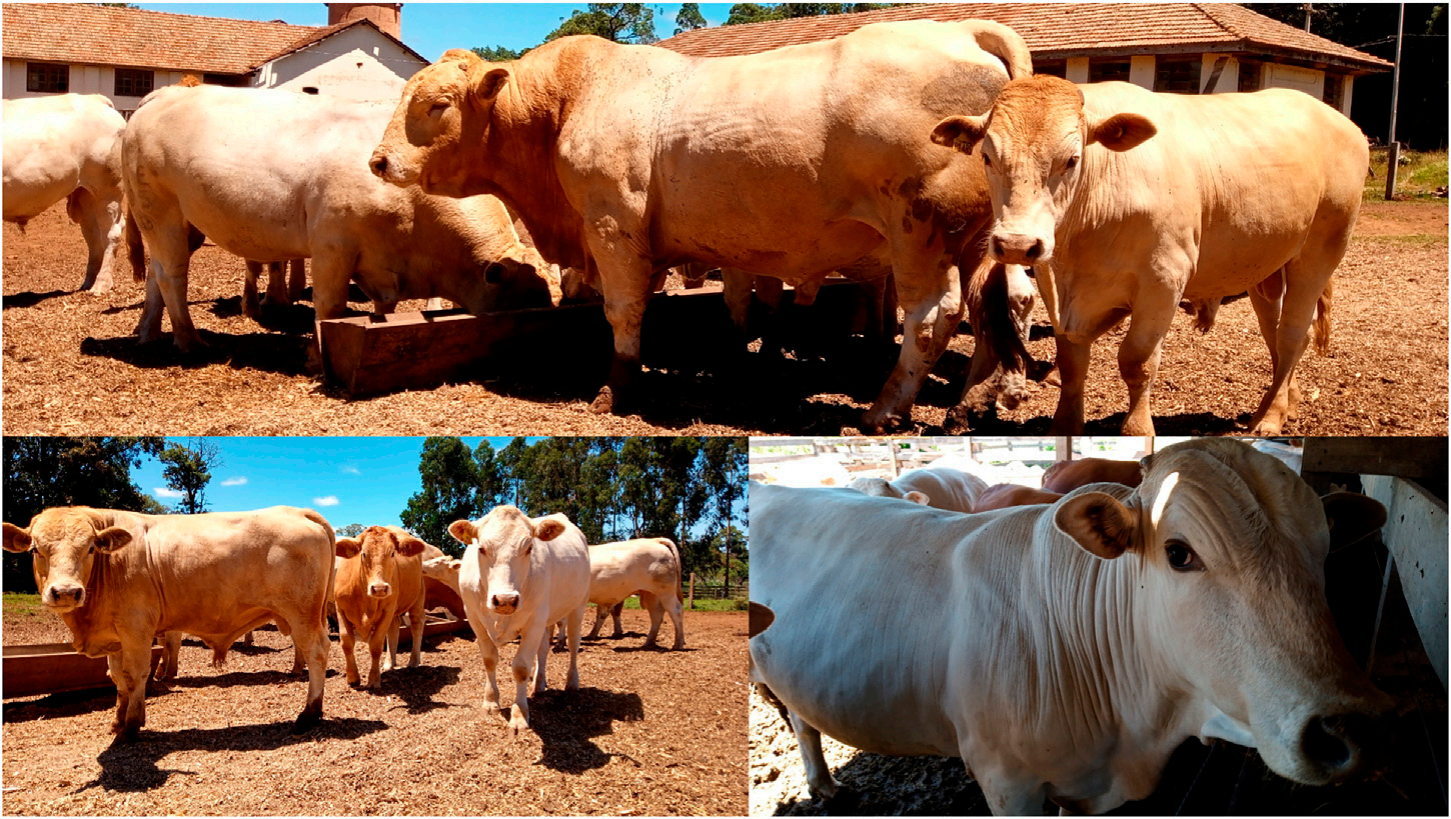
Purunã animals from the Agronomic Institute of Paraná (IAPAR, Ponta Grossa, Parana, Brazil).

Based on the first results, IAPAR researchers conducted a second mating to generate another set of animals using the progenies resulting from the previous F1 population. The goal at that point was to combine all favorable characteristics in a composite that presented a heavyweight and produce a high-quality carcass. The hypothesis for using the breeds mentioned earlier was to capture a particular contribution from each breed to create a composite population with higher productive performance and adapted to the tropical and subtropical regions of Brazil. The Angus breed provided traits related to precocity, more docile temperament, and high meat quality (Cristiana and Mirela, 2018; Taye et al., 2018); Charolais provided a higher weight gain and carcass yield (Jahuey-Martínez et al., 2019), and finally, Caracu and Canchim contributed with rusticity, heat tolerance, and parasite resistance (Urbinati et al., 2016; Pires et al., 2021). Such animals are very well adapted to tropical environmental conditions and showed good potential to gain weight (Ito et al., 2010). The characterization of Purunã, defined by the Brazilian Purunã Cattle Breed Association (Ponta Grossa, Paraná, Brazil), is that the animals must present short hair with a shiny aspect, admitting variation on the coat color (red, white, black, and bay), medium-to-large size, and good muscle distribution as shown in Figure 1. Additionally, the animals are expected to be docile and prolific, with sexual precocity and fast carcass finishing.

Some studies in Brazil have evaluated the performance of the Purunã breed for carcass traits (Ito et al., 2010), meat production and quality traits (Missio et al., 2015), growth (Moura et al., 2014), and weight at different time points (Otto et al., 2021). These studies indicate animals slaughtered at 24 months of age, weighed an average of 460 kg, possessed a fat thickness close to 3 mm, and had a substantial concentration of fatty acids in the meat. In addition, estimates of genetic parameters for growth traits have demonstrated heritable estimates (0.05–0.21) for body weights measured at different ages (Otto et al., 2021). However, no previous research to date has evaluated the genetic diversity and population structure of the Purunã breed. Although the crossbreeding increases the genetic variability of a population, their development history and population management across generations could have impacted the genetic diversity of the population formed (Peripolli et al., 2020). Genetic diversity studies play an important role in the constitution of a crossbreeding program since how the variability is controlled may interfere with the heterosis produced and impact the expected hybrid vigor.

Genetic diversity studies are crucial in the initial phase, called pre-breeding, in which it is possible to regenerate, characterize, explore, and promote the conservation of variability of the population (Pontes et al., 2020). The parameters estimated include inbreeding coefficients of the individual animals, the genetic relationship between animals, and overall levels of homozygous and heterozygous regions in the genome as well as their distribution along the chromosomes (Biscarini et al., 2020). Furthermore, linkage disequilibrium needs to be estimated for better implementing genomic selection and for identifying conserved segments of the genome among breeds (Larmer et al., 2014). All these metrics contribute to a better understanding of the genetic events that happen in the population, the impact of decisions made in the past, and the strategies that will be taken in the future. Our goal with this study was to characterize the genetic and genomic diversity and the population structure of a new composite beef cattle breed—Purunã, based on genomic and pedigree information.

## 2 Material and Method

One hundred animals of the Purunã breed were randomly sampled and genotyped using the GGP Bovine 100K array (GGP, 2021) containing over 100,000 single nucleotide polymorphisms (SNPs). The genetic material was provided by the Agronomic Institute of Paraná (IAPAR, Ponta Grossa, Paraná, Brazil). For the genotype quality control (QC), only autosomal chromosomes were retained and a QC was performed separately for each analysis. For runs of homozygosity (ROH) and heterozygous-enriched regions (HER), we removed SNPs with call rate lower than 0.90, duplicated position, non-autosomes, or without a known position (Ferenčaković et al., 2013; Biscarini et al., 2020). For the other analyses, minor allele frequency (MAF <0.05) and extreme departure from the Hardy-Weinberg equilibrium (HWE <10^–6^) parameters were also used to filter out SNPs.

For the pedigree database, information from 11,205 animals born between 1997 and 2019 was considered, where 5,224 were males and 5,981 females, and the base population was formed by 3,999 animals. These data were used to create the pedigree database including information on individual animals, sire, dam, sex, and birth date.

### 2.1 Population Stratification

#### 2.1.1 Principal Component Analysis

To assess the similarities between the Purunã breed and Angus, Canchim, Charolais, and Nellore breeds, we performed a principal component analysis (PCA) by PLINK v1.9 software (Purcell et al., 2007). The genotypes of the Angus, Charolais, and Canchim were retrieved from the WIDDE database (Sempéré et al., 2015), and the Nellore breed genotypes were provided by the Katayama Agropecuaria Ltda breeding company. The PCA was estimated based on the standardized variance of the genomic relationship matrix (**G**) where the covariance of each SNP was divided by the respective variance, using only the SNPs in common for all breeds (after the QC), as the following equation proposed by (VanRaden, 2008):

$$G=\frac{\left(M-2P\right)\left(M-2P\right)\prime }{2\text{Σ}p_{i}\left(1-p_{i}\right)},$$ (1)

where **M** is a matrix of counts of allele A, p_i_ is the frequency of allele A of *i*th SNP, and **P** is a matrix with each row containing the p_i_ values.

#### 2.1.2 Admixture Analysis

The admixture analysis was performed using the ADMIXTURE software (Alexander et al., 2015) to assess the evolutionary history between the Purunã breed and its founder breeds (Angus, Charolais, Canchim, and Nellore). This analysis estimates ancestries by efficiently computing maximum likelihood estimates in a parametric model as (Alexander and Lange, 2011):

$$ℒ\left(Q,F\right)=\underset{ij}{\sum }\left\{n_{ij}|np_{ij}+\left(2-n_{ij}\right)|n\left(1-p_{ij}\right)\right\},$$ (2)

where p_ij_ is the success probability in the binomial distribution n_ij_ ∼ Bin(2, p_ij_) depending on the fraction q_ik_ of i’s ancestry attributable to population *k* and on the frequency f_*k*j_ of allele 1 in the population *k.* The matrices **Q** = (q_ik_) and **F** = (f_kj_).

The success of the analysis is dependent on the correct choice of *K*, which represents the number of ancestral populations. We evaluated K equal to 1 until 20, but only K = 2 and 3 were chosen to be shown here, which have more biological interpretation and K = 3 had the smallest cross-validation error. The “pong” package (Behr et al., 2016) was used to cluster the results and visualize the population structure.

#### 2.1.3 Phylogenetic Tree

To estimate the distance among the populations, we used the hapFLK software (Fariello et al., 2013) based on the approach described by Bonhomme et al. (2010). The neighbor-joining tree was built from the Reynolds’ genetic distances (Reynolds et al., 1983) between pairs of populations. Reynold’s distance was estimated using the co-ancestry coefficient, where this coefficient is the probability that a random pair of genes at the same locus within a randomly chosen population is identical-by-descent, providing a natural measure of genetic drift. It is assumed that the allele frequency is equal to

$${\hat{p}}_{0}=\frac{1\prime n{ℱ}^{-1}p}{1\prime n{ℱ}^{-1}1_{n}},$$ (3)

where *p* is the frequency, ℱ is the co-ancestry matrix, and ${\hat{p}}_{0}$ is the unbiased linear estimate with minimum variance, with 1′n denoting the n-vector made of 1’s.

### 2.2 Population Structure

#### 2.2.1 Inbreeding Metrics

Six models of inbreeding coefficient estimates were analyzed. The first model was based on pedigree information (F_PED_), using the ENDOG v4.8 software (Gutiérrez et al., 2010), following the method proposed by Meuwissen and Luo (1992) in which the average F of a given generation t (F_t_) was calculated as follows:

$$F_{t}=1-{\left(1-\text{Δ}F\right)}^{t},$$ (4)

in which ΔF is the change in the inbreeding rate from one generation to another, as the following equation:

$$\text{Δ}F=\frac{\left(F_{t}-F_{t-1}\right)}{\left(1-F_{t-1}\right)},$$ (5)

in which F_t_ and F_t-1_ represent the average inbreeding estimates for the current and the previous generation (Falconer and Mackay, 1996).

The second method was based on the homozygous genotypes observed and expected (F_HOM1_), calculated as follows (Purcell et al., 2007):

$$F_{HOM1}=\frac{H_{exp}-H_{obs}}{H_{exp}},$$ (6)

where $H_{exp}$ is the expected value (proportion) for homozygous genotypes, and $H_{obs}$ is the observed value for the homozygous genotypes.

The third method was based on genotype additive variance (F_GRM_), using the following model (VanRaden, 2008):

$$F_{GRM}=\frac{{\left[x_{i}-2p_{i}\right]}^{2}}{h_{i}-1}\text{in which }h_{i}=2p_{i}\left(1-p_{i}\right),$$ (7)

where x_i_ is the number of reference allele copies of the *i*th SNP, and p_i_ is the reference allele frequency in the population. Similar to the second method, the methodology F_HOM2_ was based on homozygous genotypes following the model:

$$F_{HOM2}=1-\frac{x_{i}*\left(2-x_{i}\right)}{h_{i}}.$$ (8)

The aforementioned models are all dependent on the genotype allele frequency, and for this reason, a fifth model was a test based on the correlation between uniting gametes (F_UNI_) using the following model Yang et al. (2010):

$$F_{UNI}=\frac{\left[x_{i}^{2}-\left(1+2p_{i}\right)*x_{i}+2p_{i}^{2}\right]}{h_{i}}.$$ (9)

The last method was based on the sum of ROH individual length divided by the total length of the autosomal genome (F_ROH_) using the following equation (McQuillan et al., 2008):

$$F_{ROH}=\frac{\sum_{i=i}^{n}f\left(ROH_{i}\right)}{\sum_{j=1}^{A}h\left(j\right)},$$ (10)

where $f\left(ROH_{i}\right)$ is the ROH length of individual *i*th, *n* is the total number of homozygous genomic regions of each individual, *h*(*j*) is the length of chromosome *j*th, and *A* is the number of autosomal chromosomes (A = 29). Still, for each class of ROH (<2 Mb, 2–4 Mb, 4–8 Mb, 4–16 Mb, >16 Mb, <8 Mb, and >8 Mb), inbreeding estimates were obtained by dividing the total sum of ROH segments by the total length of the cattle autosomal genome covered by SNPs. All the genomic inbreeding coefficients were calculated using the PLINK v1.9 software (Purcell et al., 2007). The PROC CORR option of the SAS statistical software (SAS Institute Inc., 2013) was used to correlate the inbreeding coefficient estimates. A heatmap was created for better visualization of the results through the “plotly” package (Sievert, 2020).

#### 2.2.2 Linkage Disequilibrium

The linkage disequilibrium (*r* ^2^) was estimated by PLINK v1.9 software. To observe the *r* ^2^ decrease along with the increase in the marker distance, we used the binning approach estimating the *r* ^2^ average of each distance from 10 to 100 kb in each 10 kb, and after the distance of 100 kb in each 100 kb until the distance of 1,000 kb (1 Mb). As a preliminary analysis, we defined that the bins reported in this study were required to have at least 50 pairwise markers to estimate the binned average of *r* ^2^.

#### 2.2.3 Effective Population Size

Two methodologies were used to estimate the effective population size (Ne). The first method used pedigree information through the following equation:

$$Ne=\;\frac{1}{2}\text{Δ}F,\mathrm{Where}\text{Δ}F=\frac{\left(F_{t}-F_{t-1}\right)}{\left(1-F_{t-1}\right)},$$ (11)

where F_t_ and F_t−1_ are the average inbreeding of offspring and their parents, respectively (Falconer and Mackay, 1996). The estimate was performed using the POPREP software (Groeneveld et al., 2009).

The second method was performed using genomic information, and investigated with the relationship method between LD variances and Ne through the following formula (Corbin et al., 2012):

$$N_{e\left(T\right)}=\left(4f{\left(c_{t}\right)}^{-1}\left(E{\left[r^{2|ct}\right]}^{-1}-\alpha \right)\right),$$ (12)

where Ne is the effective population size at the *t*th generation, c_t_ is the recombination rate for the physical distance between the markers, α is the probability for the occurrence of mutation, and *r* ^2^ is the LD value.

#### 2.2.4 Consistency of the Gametic Phase

The consistency of the gametic phase (CGP) was taken by the square root of *r* ^2^ values adding the sign from the disequilibrium metric (D), as:

D=p(ab)−p(a)p(b), (13)

where *p(a)* is the frequency of the haplotype-a, *p(b)* is the frequency of the haplotype-b, and *p*_*ab*_ is the haplotype frequency with allele *a* on the first locus and allele *b* on the second locus. The CGP was assumed as the Person correlation between each founder breed and Purunã using the signed-squared-root values. To estimate the CGP, only the SNPs in common (after the quality control) between each breed pair were used to estimate the CGP based on the same distance and bin described in the LD section.

### 2.3 Proportion of Polymorphic SNPs and Distributions of SNPs by the MAF Range

The proportion of polymorphic SNPs, after QC, was calculated based on the MAF. The distributions of SNPs were calculated on 10 MAF ranges from 0 to 0.5 defined every 0.05 points in MAF.

### 2.4 Runs of Homozygosity

The PLINK v1.9 software was used for the ROH identification based on the following criteria:

• One heterozygous and one missing SNP were allowed;
• The window of the threshold used was 0.05;
• The gap between consecutive SNPs could not be higher than 1,000 kb;
• The minimum length of an ROH was 500 kb;
• The minimum number of consecutive SNPs that create an ROH must be equal to or greater than 30;
• The density of 1 SNP used in at least 50 kb;
• A sliding genomic window was used with 50 SNPs.

ROHs were classified in the following classes: <2 Mb, 2–4 Mb, 4–8 Mb, 4–16 Mb, and >16 Mb (Lozada-Soto et al., 2021; Mulim et al., 2022). A region found in 36% of the population was considered for future analysis (functional and phylogenetic analysis).

### 2.5 Heterozygosity-Enriched Regions

The detectRUNS package (Biscarini et al., 2019) was used for the detection of HER following the consecutive-SNPs method. For the SNPs’ consecutive analysis, the following parameters were considered:

• a minimum number of 20 consecutive SNPs constitutes an HER;
• a minimum length of 500 kb;
• a minimum of two homozygous and one missing SNP is allowed; and
• the maximum gap between consecutive SNPs could not be higher than 1,000 kb.

The genomic regions that showed at least 10% of the animals with HER were included in the subsequent functional analyses and phylogenetic tree.

### 2.6 Functional Analyses

The genomic regions considered as ROH and HER islands were used for genomic annotations. The GALLO package (Fonseca P. A. S. et al., 2020) was used for the annotation of genes in these regions, with the annotated data for *Bos taurus* from the Ensembl database (www.ensembl.org/Bos_taurus/Info/Index), version ARS-UCD1.2 (Rosen et al., 2020). Subsequently, the WebGestaltR package (Wang et al., 2020) was used for the Gene Ontology (GO) analyses to identify biological processes, molecular functions, and cellular components in which the positional candidate genes are involved in.

## 3 Results

### 3.1 Population Stratification

#### 3.1.1 Principal Component Analysis

**FIGURE 2:**
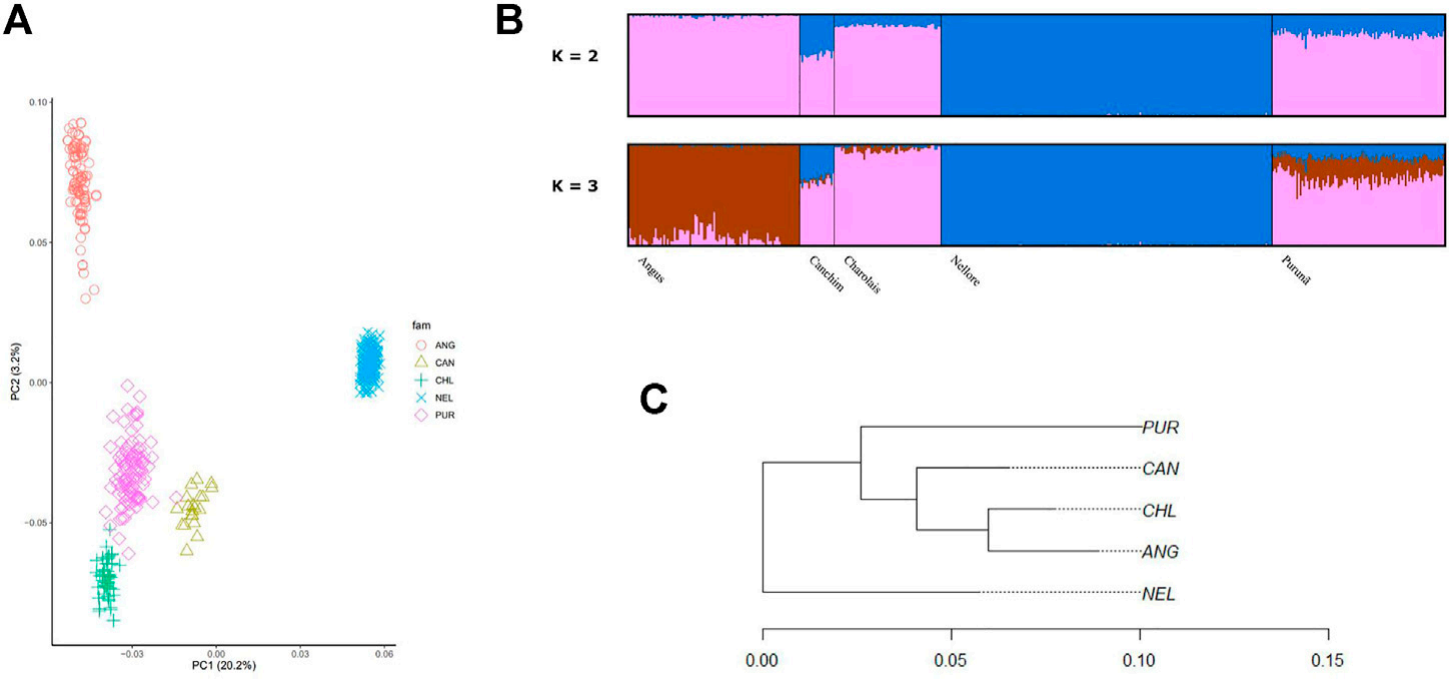
Population stratification of the Purunã breed. **(A)** Principal component analysis (PCA) including Purunã, Angus, Canchim, Charolais, and Nellore breed animals. **(B)** Admixture analysis of Purunã, Angus, Canchim, Charolais, and Nellore breeds. **(C)** Phylogenetic tree using Reynold’s distance for the Purunã (PUR), Angus (ANG), Canchim (CAN), Charolais (CHL), and Nellore (NEL) populations.

The PCA among the populations of Purunã, Angus, Canchim, Charolais, and Nellore is presented in Figure 2A. The first principal component (PC1) explained 20.2% of the variation among the populations, while the second principal component (PC2) accounted for 3.2%. As shown in Figure 2A, the animals are grouped within breeds, with no clear mixture between groups, even for composite populations such as Purunã. The breeds closer to the Purunã are Charolais, Canchim, and Angus.

#### 3.1.2 Admixture Analysis

Figure 2B presented the admixture analysis for Purunã, Angus, Canchim, Charolais, and Nellore populations for K = 2 and 3. For K = 2, two groups were observed and the mixture between them indicates that two distinct founder populations (*Bos taurus taurus* and *Bos taurus indicus*) were used when developing the Purunã breed. In average, Angus had 99.2% and 0.9%, Canchim 60.6% and 39.4%, Charolais 89.6% and 10.4%, Nellore 99.8% and 0.2%, and Purunã had 80.8% and 19.2% from ancestral population 1 and 2, respectively. For K = 3, three groups were observed to affect the admixture analysis for the population in the study. This result (K = 3) indicates more contribution from Charolais and Canchim in the Purunã breed, following the Angus breed and a small proportion of the Nellore breed.

#### 3.1.3 Phylogenetic Tree

Figure 2C shows the genomic population tree for the breeds Purunã (PUR), Angus (ANG), Charolais (CHL), Canchim (CAN), and Nellore (NEL). There is a division into groups on the tree but the distance from one group to another is not high (0.05). Nellore appears in one section while Purunã, Canchim, Charolais, and Angus are situated in three other nodes, grouping in accord with clades of breed proximity.

### 3.2 Population Structure

#### 3.2.1 Inbreeding

The averages of inbreeding coefficients are presented in Table 1.

**TABLE 1:** Inbreeding coefficient estimates with different methodologies for animals of the Purunã breed.

	N	Mean	Std Dev	Minimum	Maximum
**F**_**PED1**_	11,205	0.002	0.019	0.000	0.375
**F**_**PED2**_	100	0.007	0.023	0.000	0.125
**F**_**HOM1**_	100	−0.009	0.027	−0.052	0.163
**F**_**GRM**_	100	−0.009	0.041	−0.092	0.095
**F**_**HOM2**_	100	−0.009	0.032	−0.060	0.171
**F**_**UNI**_	100	−0.009	0.023	−0.052	0.133
**F**_**ROH**_	100	0.029	0.024	0.004	0.190
**F**_**< 2MB**_	100	0.004	0.002	0.000	0.010
**F**_**2-4MB**_	100	0.007	0.003	0.001	0.023
**F**_**4-8MB**_	100	0.007	0.005	0.000	0.021
**F**_**8-16MB**_	100	0.006	0.007	0.000	0.032
**F**_**> 16MB**_	100	0.005	0.016	0.000	0.127
**F**_**< 8MB**_	100	0.018	0.007	0.002	0.045
**F**_**> 8MB**_	100	0.011	0.020	0.000	0.145

N: number of individuals analyzed; Mean: average of inbreeding coefficient; Std Dev: standard deviation.

F_PED1_: inbreeding coefficient based on the pedigree for all individual in the Purunã breed.

F_PED2_: inbreeding coefficient based on the pedigree for Purunã genotyped individuals.

F_HOM1_: inbreeding coefficient based on the number of observed and expected homozygous genotypes.

F_GRM_: inbreeding coefficient based on additive genotypic variance.

F_HOM2_: inbreeding coefficient based on homozygosity of genotypes.

F_UNI_: inbreeding coefficient based on the correlation between uniting gametes.

F_ROH_: inbreeding coefficient based on the length of the ROH’s and the total length of the autosomal genome.

**FIGURE 3:**
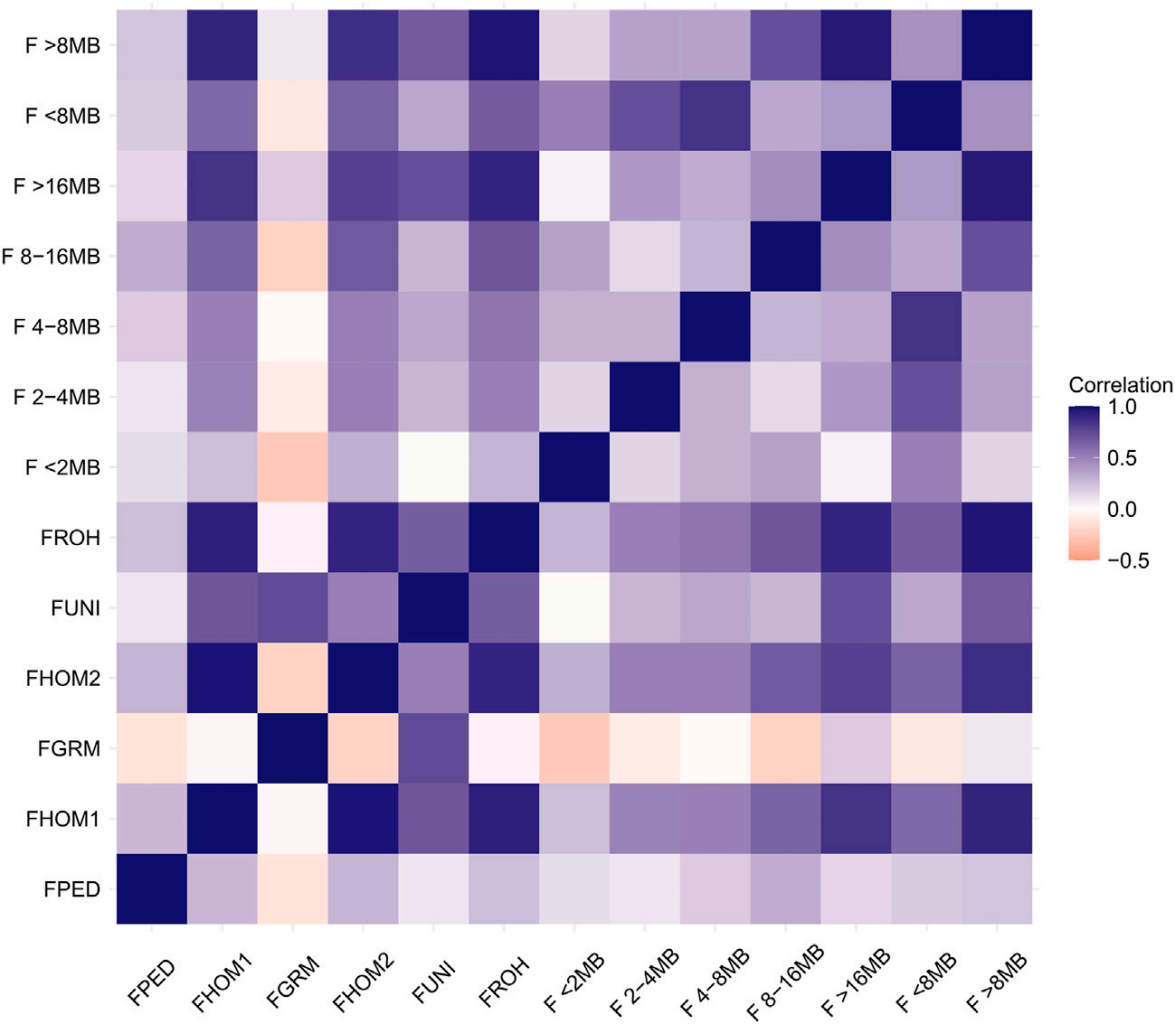
Correlation among inbreeding estimation methods.

The average for the inbreeding coefficient estimated based on pedigree for all Purunã individuals (F_PED2_) was 0.002. The methods F_HOM1_, F_HOM2_, F_UNI_, and F_GRM_ were the methods showing the lowest average values (−0.009), while the highest inbreeding coefficient average was obtained by the F_ROH_ metric (0.029). The correlations among the inbreeding coefficients method are presented in Figure 3.

Strong correlations were found between the methods: F_HOM1_-F_HOM2_ (0.97), F_HOM1_-F_ROH_ (0.93), F_HOM1_-F_>16MB_ (0.85), F_HOM1_-F_>8MB_ (0.89), F_HOM2_-F_ROH_ (0.90), F_HOM2_-F_>16MB_ (0.79), and F_HOM2_-F_>8MB_ (0.85). Low values were found for all correlations among F_PED_ and the other methods. The F_GRM_ method shows a very weak correlation for almost all the methods, except for the F_UNI,_ when the correlation was classified as moderate (0.74). Negative correlations were found for the methods: F_GRM_-F_HOM2_ (−0.21), F_GRM_-F_<2MB_ (−0.24), F_GRM_-F_2–4MB_ (−0.08), F_GRM_-F_4–8MB_ (−0.01), F_GRM_-F_8–16MB_ (−0.21), F_GRM_-F_<8MB_ (−0.11), and F_GRM_-F_PED_ (−0.12).

#### 3.2.2 Linkage Disequilibrium

The average LD ranged from 0.43 to 0.04, with a distance between two markers of 10 to 1,000 kb, respectively. The general average of LD was 0.031 (±0.0517) at the average distribution of the markers 4.856 (±2.8890) Mb. The decrease in LD with an increase in the marker distance can be observed in Supplementary Figure S1.

#### 3.2.3 Effective Population Size

The effective population size based on pedigree was 158 for the current generation. On the other hand, the genomic-based Ne differed based on the generation and software used. The SNeP software (Barbato et al., 2015) and the PLINK software enabled the estimation of Ne up to the 13th and 5^th^ generation back, respectively. For the SNeP, in the 13th generation, the Ne was 229, while for the PLINK, the result for the same generation was 207. The Ne estimated for the 5^th^ generation on PLINK was equal to 115.

#### 3.2.4 Consistency of the Gametic Phase

The consistency of the gametic phase between Purunã and Angus, Canchim, Charolais, and Nellore is presented in Table 2.

**TABLE 2:** Consistency of the gametic phase based on Pearson correlation, between the Purunã breed and its founder breeds: Angus, Canchim, Charolais, and Nellore breeds.

Distance (kb)	Angus	Canchim	Charolais	Nellore
20	0.40	0.42	0.43	0.27
30	0.36	0.40	0.42	0.18
40	0.30	0.36	0.41	0.17
50	0.29	0.33	0.39	0.14
60	0.29	0.33	0.39	0.13
70	0.26	0.33	0.35	0.11
80	0.24	0.30	0.33	0.08
90	0.24	0.30	0.33	0.04
100	0.20	0.27	0.31	0.04
200	0.18	0.24	0.25	0.04
300	0.12	0.20	0.18	0.03
400	0.10	0.16	0.16	0.02
500	0.10	0.13	0.12	0.02
600	0.10	0.13	0.12	0.02
700	0.09	0.11	0.11	0.01
800	0.09	0.11	0.10	0.01
900	0.08	0.11	0.10	0.00
1,000	0.07	0.09	0.10	0.00

The highest correlation, at 20 kb between SNP pairs, between Purunã and the other breeds was found with Charolais (0.43), followed by Canchim (0.42), Angus (0.40), and Nellore (0.27). The distance of 10 kb showed a lower number of pairwise markers than the threshold (<50) used as a criterion. Therefore, these results were not presented.

### 3.3 Proportion of Polymorphic SNPs and Distribution of SNPs by MAF Range

The proportion of polymorphic SNPs based on the MAF category were as follows: MAF_0.00-0.05_ 3,232 (3.67%); MAF_0.05-0.10_ 2,967 (3.37%); MAF_0.10-0.15_ 4,242 (4.82%); MAF_0.15-0.20_ 5,792 (6.59%); MAF_0.20-0.25_ 7,255 (8.25%); MAF_0.25-0.30_ 9,314 (10.59%); MAF_0.30-0.35_ 11,287 (12.83%); MAF_0.35-0.40_ 13,283 (15.10%); MAF_0.40-0.45_ 14,932 (16.98%); and MAF_0.45-0.50_ 15,652 (17.80%).

### 3.4 Runs of Homozygosity

**FIGURE 4:**
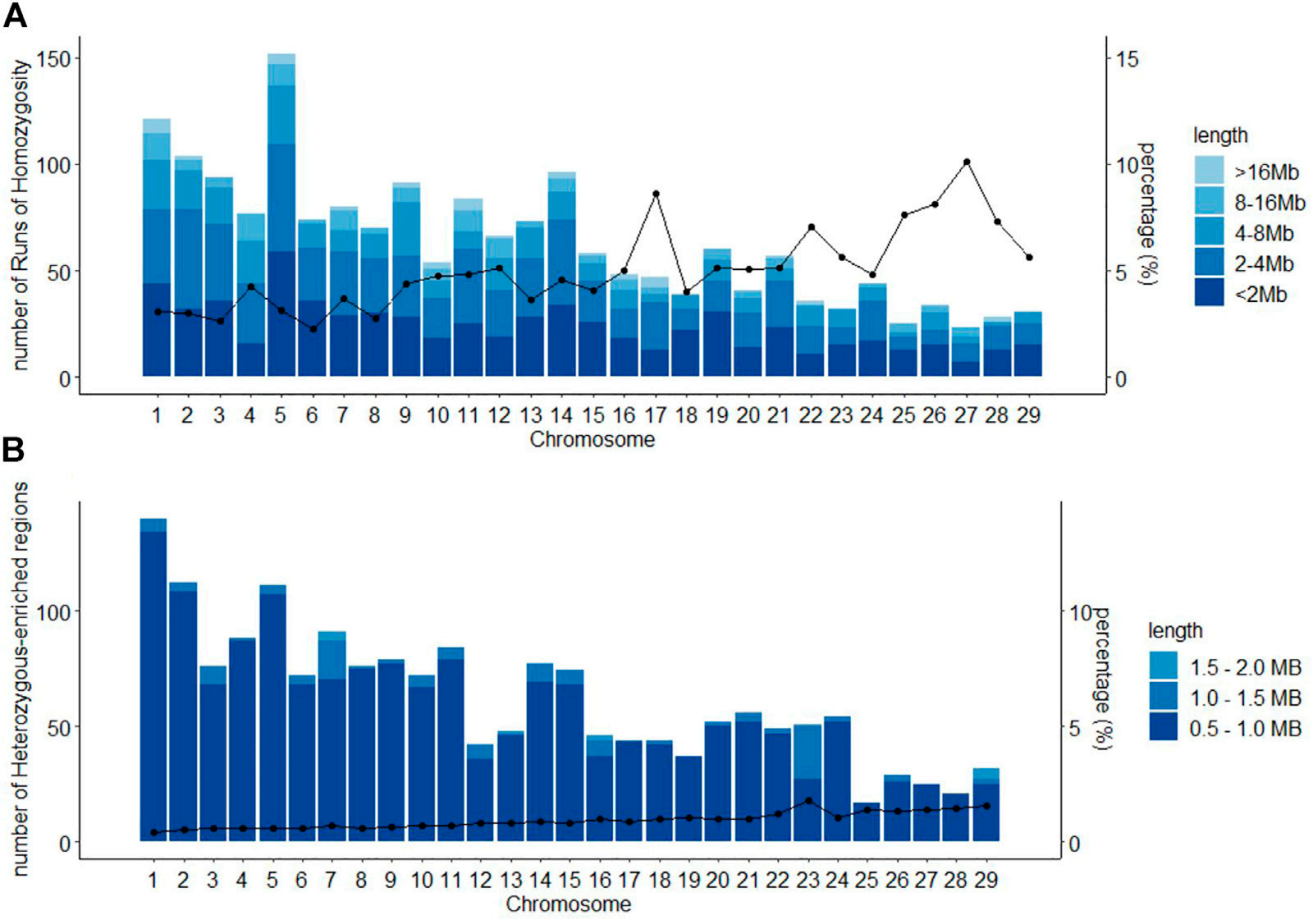
Classification of runs of homozygosity ROH **(A)** and heterozygous-enriched regions HER **(B)**, by chromosome, according to the length size in the Purunã breed, and the average percentage of chromosome covered by ROHs/HERs.

A total of 1,839 ROHs were found for the Purunã breed. The distribution along all autosomal genomes can be observed in Figure 4A and the ROH length size division. The length of ROH observed here can be classified as 37.4% for <2 Mb; 25.3% as 2–4 Mb; 17.1% as 4–8 Mb; 7.6% as 8–16 Mb; and only 2.6% ROH greater than 16 Mb. The chromosome that presented the highest amount of ROHs was the BTA5, followed by the BTA1, where the concentration of ROHs >16 Mb was superior compared to all other autosomes. The chromosomes that showed the smallest number of ROHs were the BTA27 and BTA25, representing a small fraction of regions in ROH.

### 3.5 Heterozygous-Enriched Regions

In total, 1,799 HERs were found in the Purunã breed. The HER pattern distribution along with all autosomal genomes is shown in Figure 4B. Around 92.3% of the HERs found were classified in the length of 0.5–1.0 Mb; 7.0% as 1.0–1.5 Mb; and 0.7% as 1.5–2.0 Mb. No HER greater than 2 Mb was found for Purunã. The chromosome that presented the highest amount of HER was the BTA1, while BTA25 had the smallest number of HER.

### 3.6 ROH and HER Islands and Functional Analyses

#### 3.6.1 Runs of Homozygosity

With the ROH analysis, we found a common region in homozygosity present in 36% of the animals, despite the fact that Purunã is a recently-developed composite breed. This region is located on BTA5 between 54, 304, 681 bp and 62, 031, 799 bp, and has a length of 7.73 Mb, where 131 SNPs are present in this region. This region is responsible for coding 220 genes, with 181 protein-coding genes, seven pseudogenes, five long non-coding RNA, nine microRNA, ten miscellaneous RNA, six small nucleolar RNA, and two small nuclear RNA. The list of all the genes found in this region is presented in Supplementary Table S1. The significant Gene Ontology (GO) terms (*p* < 0.05) in which these genes are part of are presented in Table 3.

**TABLE 3:** Significant (*p* < 0.05) Gene Ontology (GO) terms for the genes located within runs of homozygosity regions in the Purunã breed.

	Description	*p*-Value	Genes
*Biological process*			
GO:0,043,648	Dicarboxylic acid metabolic process	0.001	*SHMT2; GLS2; AMDHD1; HAL*
GO:0,007,600	Sensory perception	0.004	*MY O 1A; MIP; RDH5; OR10P1; ENSBTAG00000047825; ENSBTAG00000046778; ENSBTAG00000048295; ENSBTAG00000002913; OR10A7; ENSBTAG00000037629*
GO:0,043,473	Pigmentation	0.008	*DCTN2; PMEL; CD63*
GO:0,006,520	Cellular amino acid metabolic process	0.014	*MARS1; SHMT2; GLS2; AMDHD1; HAL*
GO:0,006,091	Generation of precursor metabolites and energy	0.015	*NDUFA4L2; SHMT2; PTGES3; CS; COQ10A; BLOC1S1*
GO:0,044,282	Small molecule catabolic process	0.019	*CYP27B1; SHMT2; GLS2; AMDHD1; HAL*
GO:0,051,606	Detection of the stimulus	0.022	*OR10P1; ENSBTAG00000047825; ENSBTAG00000046778; ENSBTAG00000048295; ENSBTAG00000002913; OR10A7; ENSBTAG00000037629*
GO:0,007,422	Peripheral nervous system development	0.023	*NAB2; ERBB3*
GO:0,006,766	Vitamin metabolic process	0.041	*CYP27B1; SHMT2*
GO:0,009,991	Response to the extracellular stimulus	0.047	*CYP27B1; DDIT3; MARS1; SLC39A5*
***Molecular function***			
GO:0,004,984	Olfactory receptor activity	0.005	*OR10P1; ENSBTAG00000047825; ENSBTAG00000046778; ENSBTAG00000048295; ENSBTAG00000002913; OR10A7; ENSBTAG00000037629*
GO:0,016,741	Transferase activity, transferring one-carbon groups	0.018	*EEF1AKMT3; METTL1; SHMT2; METTL7B*
GO:0,000,049	tRNA binding	0.032	*METTL1; MARS1*
***Cellular component***			
GO:0,009,295	Nucleoid	0.017	*SHMT2; ATP5F1B*
GO:0,016,328	Lateral plasma membrane	0.018	*MY O 1A; ERBB3*
GO:0,045,177	Apical part of the cell	0.022	*MY O 1A; MIP; ERBB3; NEDD1*
GO:0,005,759	Mitochondrial matrix	0.031	*TSFM; SHMT2; ATP5F1B; CS; BLOC1S1*
GO:0,098,687	Chromosomal region	0.034	*DCTN2; MBD6; NABP2; CDK2*
GO:0,005,788	Endoplasmic reticulum lumen	0.045	*OS9; RDH5*

**FIGURE 5:**
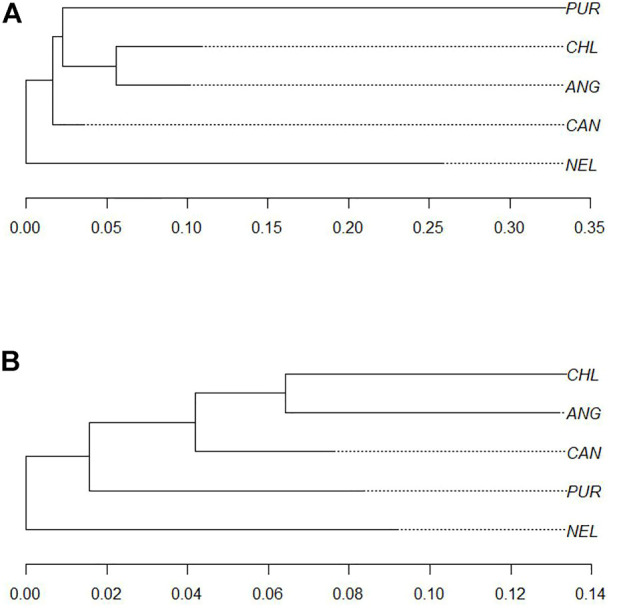
Phylogenetic trees. **(A)** Phylogenetic tree for homozygous regions comparing the Purunã (PUR), Angus (ANG), Canchim (CAN), Charolais (CHL), and Nellore (NEL) populations. **(B)** Phylogenetic tree for growth pathway in heterozygous-enriched regions comparing the Purunã (PUR), Angus (ANG), Canchim (CAN), Charolais (CHL), and Nellore (NEL) populations.

Ten biological processes, three molecular functions, and six cellular components were identified in the significant pathways. Interestingly, pathways linked to animal behavior were found in this region, including sensory perception (GO:0007600), detection of stimulus (GO:0051606), response to extracellular stimulus (GO:0009991), olfactory receptor activity (GO:0004984), and others. To track the origin of this homozygous region in Purunã, we performed a phylogenetic tree analysis, using only the SNPs allocated in this region. Figure 5A shows the phylogenetic tree for the homozygous region found in BTA5. In this particular region, the breeds Purunã, Charolais, and Angus are closer together in comparison to the Canchim and Nellore breed, indicating that Charolais and Angus might have contributed to key behavioral characteristics observed in the Purunã breed.

#### 3.6.2 Heterozygous-Enriched Regions

For the HER analysis, the regions identified in at least 10% of the animals were considered as HER islands and used to verify the candidate genes and pathways. Table 4 presents the HER island found in the Purunã breed.

**TABLE 4:** Heterozygous-enriched regions (HER) which appear in at least 10% of Purunã individuals.

CHR	%	BP1	BP2	nSNP	Length
BTA1	11	26,505,838	29,555,484	22	3,049,646
BTA2	17	42,384,465	43,575,039	23	1,190,574
BTA3	10	8,435,805	9,838,978	23	1,403,173
BTA5	14	70,752,944	72,012,890	23	1,259,946
BTA5	14	75,043,240	75,983,135	26	939,895
BTA6	11	27,154,761	28,275,511	21	1,120,750
BTA7	13	8,562,310	10,432,630	24	1,870,320
BTA10	11	44,820,482	46,032,038	25	1,211,556
BTA11	14	67,243,961	69,096,131	22	1,852,170
BTA12	10	40,237,435	41,970,427	25	1,732,992
BTA14	17	24,167,298	25,953,073	24	1,785,775
BTA14	16	50,608,626	51,640,291	23	1,031,665
BTA15	10	1,215,097	1,819,862	30	604,765
BTA18	10	23,515,690	24,470,198	23	954,508
BTA19	12	34,233,799	35,283,135	25	1,049,336
BTA20	10	44,099,958	45,220,153	21	1,120,195
BTA22	10	48,961,009	52,638,988	21	3,677,979
BTA23	27	26,021	1,697,122	27	1,671,101
BTA24	10	40,975,659	41,855,725	21	880,066

CHR: chromosome.

%: percentage of the population that presented this island.

BP1: position in the base pair where the HER start.

BP2: position in the base pair where the HER end.

nSNP: number of SNPs, that HER covers.

Length: HER, length.

We found 19 HERs distributed in 17 chromosomes, where the BTA5 and BTA14 presented two HERs in each chromosome. The most frequent HER (27% of the population) was found in BTA23. The longest HER was found in BTA22 with a length size of 3.67 Mb, and the smallest HER was found in the BTA15 at 0.60 Mb.

All these regions are responsible for coding 413 genes, including 363 protein-coding, six pseudogenes, 13 long non-coding RNAs, seven microRNAs, two miscellaneous RNAs, one small nucleolar RNA, 15 small nuclear RNAs, three processed pseudogenes, and three ribosomal RNAs. The list of all the genes found in these regions is presented in Supplementary Table S2. The significant GO terms (*p* < 0.05) and their related genes are presented in Table 5.

**TABLE 5:** Significant (*p* < 0.05) Gene Ontology (GO) terms, to biological process, to heterozygous-enriched regions found in the Purunã breed.

	Description	*p*-value	Genes
*Biological process*			
GO:0,051,270	Regulation of cellular component movement	0.003	*SLAMF1; RAC2; ZNF609; SDCBP; FLCN; MAP2K3; IQCF1; HYAL2; HYAL1; SEMA3B; SEMA3F; MST1; DAG1; RHOA; ELP6; PTPRM*
GO:0,010,563	Negative regulation of the phosphorus metabolic process	0.012	*PWP1; ELFN2; RTRAF; FLCN; HYAL2; INKA1; DAG1; RHOA; QARS1; PRKAR2A*
GO:0,090,407	Organophosphate biosynthetic process	0.013	*CD244; PIGM; LPCAT2; PRPSAP2; PEMT; FLCN; IP6K1; IMPDH2; IPGK2; TREX1; NME6*
GO:0,072,521	Purine-containing compound metabolic process	0.018	*ATP1A2; PTGDR; PRPSAP2; SHMT1; FLCN; RHOA; IMPDH2; UQCRC1; TREX1; NME6; NDUFV2*
GO:0,040,007	Growth	0.019	*PPIB; SDCBP; MT2A; MT3; RAI1; FLCN; DCAF1; CISH; HYAL2; HYAL1; SEMA3B; SEMA3F; ARIH2; IP6K2*
GO:0,007,187	G protein-coupled receptor signaling pathway, coupled to the cyclic nucleotide second messenger	0.019	*PTGDR; GNA O 1; GRM2; GNAI2; GNAT1; PTH1R*
GO:0,055,086	Nucleobase-containing small molecule metabolic process	0.020	*ATP1A2; PTGDR; PRPSAP2; SHMT1; NT5M; FLCN; GMPPB; RHOA; IMPDH2; UQCRC1; TREX1; NME6; NDUFV2*
GO:0,031,647	Regulation of protein stability	0.038	*PEX19; PPIB; MT3; COPS3; USP4; TREX1*
GO:1,901,657	Glycosyl compound metabolic process	0.040	*PTGDR; PRPSAP2; IMPDH2; NME6*
GO:0,001,505	Regulation of neurotransmitter levels	0.046	*ATP1A2; KCNJ10; SYN3; SLC6A2; SHMT1; AMT*
GO:0,001,667	Ameboidal-type cell migration	0.048	*MAP2K3; HYAL2; HYAL1; SEMA3B; SEMA3F; RHOA; PTPRM*

In total, we found 17 significant GO terms involved in biological processes, eight in molecular functions, and six in cellular components. Interesting regions related to the growth pathways (GO:0,040,007) were found in the heterozygous-enriched regions in BTA10, BTA14, BTA18, BTA19, and BTA20, where 14 genes are acting in higher variability in the population. The genes are *PPIB* (peptidylprolyl isomerase B), *SDCBP* (syndecan binding protein), *MT2A* and *MT3* components of metallothionein, *RAI1* (retinoic acid-induced 1), *FLCN* (folliculin), *DCAF1* (DNA damage-binding protein 1), *CISH* (cytokine-inducible SH2), *HYAL1* and *HAYAL2* components of hyaluronidase, *SEMA3B* and *SEMA3F* components of semaforin, *ARIH2* (ariadne RBR E3 ubiquitin protein ligase 2), and IP6K2 (inositol hexakisphosphate kinase 2). To track the origin of these heterozygous regions in Purunã, we used a phylogenetic tree analysis. Figure 5B shows the phylogenetic tree for the regions related to the growth pathway found in the heterozygous-enriched regions.

## 4 Discussion

Our main goal in this study was to genetically characterize the Purunã breed by estimating genetic diversity and population structure parameters based on both genomic and pedigree information. This breed was developed by crossing, in the same proportion, Charolais, Canchim, Angus, and Caracu breeds, while Canchim is also a composite breed that has Charolais and Nellore as the main founder breeds. Therefore, the average genetic proportion for Purunã is 13/32 Charolais, 8/32 Caracu, 8/32 Angus, and 3/32 Nellore.

Based on the results from the population stratification section, the Purunã breed seems to be genetically closer to Charolais, Canchim, and Angus, with the highest genomic contribution from the Charolais breed (Figure 2B, K = 3). Yet, our findings indicate that Purunã is closer to the *Bos taurus taurus* than *Bos taurus indicus* breeds. This was expected due to the greater contribution of taurine breeds in the formation of the Purunã breed.

### 4.1 Inbreeding Metrics

The maintenance of low levels of inbreeding is also desirable in composite breeds, once an advantage of crossbreeding is heterosis. Such heterosis is influenced by the genetic distance between the parental breeds and the level of inbreeding in the population, which can affect the degree of heterosis retention (Peripolli et al., 2020). As shown in Table 1, the inbreeding coefficient for all the metrics estimated in this study ranged from −0.009 (±0.041) to 0.029 (±0.024). These results are expected as the Purunã breed is a recently developed composite breed. The low level of inbreeding, with more emphasis on negative values (outbreeding), indicates that the probability of the two homologous genes within an individual being identical-by-descendent is smaller than two homologous genes drawn at random from the reference population and the ancestry shared into the population is small (Wang, 2014). However, in terms of gain or loss variability to a reference base population (Villanueva et al., 2021), the values indicated that some variability had been gained (through migration or gene flow from other populations) or, in the cases where the inbreeding coefficient was positive, a slight loss of variability.

An accurate measure of F_PED_ could be expected when a complete, deep (many generations recorded) and no (or few) errors in the pedigree files. In the case of the Purunã breed, the information in the pedigree files goes to, on average, 2.35 generations. As the Purunã pedigree is shallow, the use of genomic information to estimate the inbreeding coefficients is a great alternative to access the inbreeding levels of the individuals. These inbreeding metrics, in addition, are not dependent on pedigree information, taking into account the Mendelian sampling variation (Doekes et al., 2019), the stochastic nature of recombination (Ferenčaković et al., 2013), and correcting the pedigree failure to assume that the founders of a population are unrelated (Rebelato et al., 2018). Yet, some metrics not only measure the levels of overall inbreeding but also give an estimate of when the inbreeding was created, as in the case of F_ROH_.

The F_ROH_ captures the highest level of inbreeding, especially because the F_ROH_ metric is capable of capturing both recent and more ancient inbreeding (Ghoreishifar et al., 2020). As shown in Table 1, the value for the ancient inbreeding coefficient (F < 8 MB) is higher than more recent inbreeding (F > 8 MB). Such ancient inbreeding could be provided by ancient generations in ancient mating and still be in the population passing it through generations. This division between ancient and recent inbreeding is helpful to manage the diversity in the population. As not all inbreeding is expected to be equally unfavorable, recent inbreeding is expected to have more negative effects than ancient inbreeding (Doekes et al., 2019), therefore maintaining a low level of recent inbreeding coefficient is a desirable goal. Figure 3 illustrates the correlation among the inbreeding coefficient metrics. All the metrics showed a low correlation with F_PED_. Some authors have already mentioned that the genomic inbreeding metrics are more accurate in assessing individual inbreeding (Curik et al., 2014; Marras et al., 2015; Doekes et al., 2019). This happens due to the particularities mentioned before about the pedigree estimation, but as the F_PED_, each metric used to calculate the genomic inbreeding coefficient has its specificities and captures a different type of inbreeding that was originally defined by Wright (1922) and/or Malécot (1948).

The genomic metrics vary according to the weight that each marker gets to find the **G** matrix or the allele frequency for each marker (Howard et al., 2017). This affects how the inbreeding is calculated for each individual and the correlation among the metrics. The metrics F_HOM_ and F_ROH_ weigh all the alleles equally, while the metrics F_UNI_ and F_GRM_ give more weight to rare alleles (Alemu et al., 2021). This could explain why the metrics F_HOM_ and F_ROH_, and F_GRM_ and F_UNI_ show moderate to strong correlation, while the F_GRM_ and F_HOM_ or the F_ROH_ classes had a negative correlation.

### 4.2 Linkage Disequilibrium, Effective Population Size, and Consistency of the Gametic Phase

Higher LD values were observed for markers located closer to each other and a faster decreased LD values were found as the distance between the markers increased, as observed in other crossbred or composite populations (Prieur et al., 2017; Deng et al., 2019). The extent of LD is strongly influenced by the population history, particularly in domestic animal populations, which have undergone bottlenecks during both domestication and the subsequent formation of breeds (Brito et al., 2015). Such LD is directly related to genomic selection, where the number of markers required to accurately predict breeding values depends on the LD (Larmer et al., 2014). Following the proposed equation by (McKay et al., 2007), the number of markers required for accurate genomic selection will be around 95,000 markers (2.67 GB/30 kb at LD = 0.2) for the Purunã breed. However, it is essential to highlight that for an implementation of genomic selection in Purunã, it is crucial that a sizable training population needs to be generated, to provide accurate genomic predictions of breeding values and selection.

Analysis of LD plays a central role in many areas of population genetics, including the determination of genetic maps, ascertainment of levels of recombination at the population level, and Ne estimation (D’Ambrosio et al., 2019). Based on all the metrics, the Ne estimates for Purunã are higher than 100 in the current generations, which is a threshold proposed by Meuwissen (2009) to ensure long-term population sustainability. The Ne estimate based on LD was able to be detected up to the fifth generation ago using the PLINK software (Purcell et al., 2007). We observed a slight divergence between the results from the SNeP and PLINK software, but not as high as reported by Barbato et al. (2015).

Understanding the LD levels, population structure, and CGP across breeds are crucial for implementing genomic selection (Brito et al., 2015). The CGP for all the evaluated breeds, including the Purunã, resulted in a low correlation, as shown in Table 2. These results indicate that the markers’ phase (or the phase between markers and QTL) is not consistent across breed pairs. In this context, the possible use of a multi-breed training population for genomic evaluations using these breeds (Purunã, Charolais, Canchim, and Angus) might not result in more accurate genomic breeding values. As the markers are not in the same phase across breeds, the ability to use one breed to determine the effects of SNP to aid in the selection of another population becomes less likely (Larmer et al., 2014).

### 4.3 Runs of Homozygosity

Figure 4A shows the number of ROHs found by chromosome in Purunã. The BTA5 showed a higher number of ROHs, as also observed in other beef cattle studies (Peripolli et al., 2018; Peripolli et al., 2020). The majority of ROH found (62.7%) were classified as short ROHs, and as the length of ROH is negatively correlated with the co-ancestry (Mastrangelo et al., 2018), the ROH found in Purunã were conceived in a more ancient generation. Taking the length of ROH and using the studies that estimate the ROH and correlate with the generation, as the work of Howrigan et al. (2011), the majority of ROH found in this study was created between 10 and 20 generations ago.

The ROH can be used for genome characterization and a better understanding of the implications of selection pressure (Marras et al., 2018). An interesting region was identified in the BTA5, which contains significant pathways related to behavioral traits. The first pathway was the sensory perception associated with a series of events required for an organism to receive a sensory stimulus, convert it to a molecular signal, and recognize and characterize the signal (AmiGO, 2021c). The second was the detection of a stimulus pathway related to a stimulus received by a cell or organism. This pathway converts a signal into a response to an extracellular stimulus, associating any movement, secretion, enzyme production, or gene expression in an extracellular stimulus (AmiGO, 2021a). The third pathway was olfactory receptor activity, a pathway related to combining with an odorant and transmitting the signal from one side of the membrane to the other to initiate a change in cell activity in response to the detection of smell (DeMaria and Ngai, 2010).

To track the origin of such a region, a phylogenetic tree (Figure 5A) was made to evaluate which breed could provide this region. As shown in Figure 5A, the Purunã, Charolais, and Angus animals seem to be genetically closer, and therefore, Angus and Charolais might have contributed to this region. Some studies have previously reported the same region with a high incidence of homozygous sequence (Szmatoła et al., 2019; Fabbri et al., 2021). Some interesting genes, already mentioned in the literature as candidate genes were identified in this region, as *MY O 1A* (Myosin IA) related to bovine heat-tolerance (Jia et al., 2019), *RDH5* (11-cis retinol dehydrogenase 5) associated with feed conversion (de Almeida Santana et al., 2016), and *AMDHD1* (amidohydrolase domain containing 1) related to reproduction (Moravčíková et al., 2019).

### 4.4 Heterozygous-Enriched Region

Maintaining diversity at a locus may be advantageous for fitness and could be subject to balancing or countervailing selection (Williams et al., 2016). These heterozygous-enriched regions are single nucleotide differences observed between paternal and maternal chromosomes and can reveal much about the population structure and demographic history (Santos et al., 2021). As shown in Figure 4B, the majority of HER found in this study were classified as shorter HER. Interestingly, one region is already mentioned as a conserved region for beef and dairy cattle in the BTA14 (Zhao et al., 2015). This region is variable in at least 17% of the Purunã individuals, demonstrating that even in more conservative regions, the crossbred could provide some variability to the animals.

Although some studies have shown that the majority of HER islands are related to immunity to diseases (Williams et al., 2016), survival rate, and fertility (Biscarini et al., 2020), an interesting pathway was found in our study related to growth. The growth pathway is a biological process related to the increase in size or mass of an entire organism, a part of an organism, or a cell (AmiGO, 2021b). In the case of *PPIB*, a gene used as a reference gene in studies of gene expression (Costa et al., 2013; da Costa et al., 2013) or the *SDCBP* gene, a possible candidate gene related to carcass weight in Hanwoo, a Korean native breed (Lee et al., 2013) and Montana Tropical Composite, a composite beef cattle population developed in Brazil (Grigoletto et al., 2020). Another gene mentioned as a possible candidate gene for meat quality and carcass yield was the *MT2A*, which is involved in glucocorticoid response and with metal and antioxidant biological responds (Haegeman et al., 2003). Although the *CISH* gene is directly related to insulin metabolism, Fonseca L. F. S. et al. (2020) indicated that this gene could play an essential role in marbling deposition. Our tracking of this pathway was not possible to define a unique breed responsible to provide such a region of HER to Purunã breed or even the breeds where the population is closer, as shown in Figure 5B. This means that such variability is not provided by a unique or small group of breeds, but by the mixture of the breeds used in the creation of the Purunã breed.

## 5 Conclusion

As observed in the admixture analyses, the Purunã breed received a more significant genetic contribution to its formation from Charolais, Canchim, and Angus. The inbreeding levels for Purunã were small based on multiple inbreeding metrics. Higher LD values were observed for markers with small distances and a faster decrease associated with an increase in the distance between the markers (ranging from 0.43 to 0.04 with distance of 10–1,000 Kb), indicating that a denser panel of markers is necessary to achieve higher levels of accuracy in a genomic selection of Purunã. A high Ne (>100) was observed in all metrics evaluated and the consistency of gametic phase analyses resulted in a small correlation among all breeds, which determines that a multi-breed genetic evaluation for Purunã might not be advantageous. An interesting homozygous region was found in the BTA5 with significant pathways related to behavior and genes related to traits such as heat-tolerance (*MY O 1A*), feed conversion rate (*RDH5*), and reproduction (*AMDHD1*). This could indicate a possible pressure of selection in such regions. For the heterozygosity, the number of HER was elevated, but this was expected since Purunã is a composite breed. Among HER regions, an interesting pathway related to growth was identified with higher variability, containing genes previously associated with carcass weight (*SDCBP*), meat and carcass quality (*MT2A*), and marbling deposition (*CISH*).

## Data Availability

The Purunã genotypes data are available in the OSF Repository (https://osf.io/7p6wt/). Genotypes from Angus, Canchim, and Charolais are available in the WIDDE database (http://widde.toulouse.inra.fr/widde/). The Nellore datasets presented in this article are not readily available because genotypes from databases Katayama are not publicly available but can be obtained through a reasonable request via the corresponding author. Requests to access the datasets should be directed to vbpedrosa@uepg.br.
