# Supplementary material for: Genetic and Genomic Characterization of a New Beef Cattle Composite Breed (Purunã) Developed for Production in Pasture-Based Systems
Source: Front Genet. 2022 Jul 18;13:858970. doi: 10.3389/fgene.2022.858970 (PMC9341487; doi:10.3389/fgene.2022.858970)
Supplement: Supplementary file 2 [file Table3.DOCX]

**
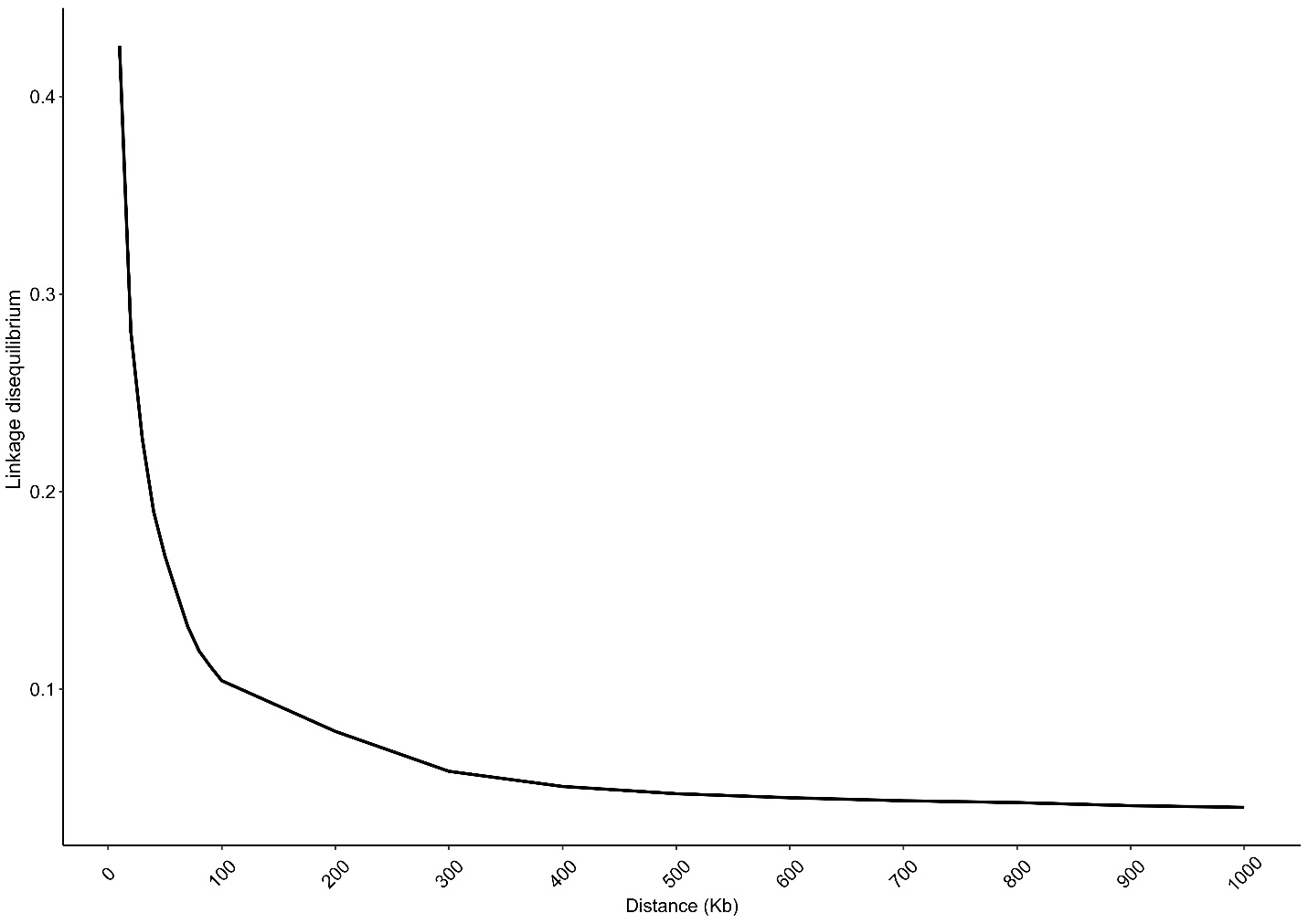
**

**Supplementary Figure 1.** Decrease of linkage disequilibrium (LD) with the increase of markers distance (kb) in the Purunã breed.
